# Frunevetmab, a felinized anti‐nerve growth factor monoclonal antibody, for the treatment of pain from osteoarthritis in cats

**DOI:** 10.1111/jvim.16291

**Published:** 2021-11-01

**Authors:** Margaret E. Gruen, Jamie A. E. Myers, Jezaniah‐Kira S. Tena, Csilla Becskei, Dawn M. Cleaver, B. Duncan X. Lascelles

**Affiliations:** ^1^ Translational Research in Pain (TRiP) Program, Department of Clinical Sciences, College of Veterinary Medicine North Carolina State University Raleigh North Carolina USA; ^2^ Behavioral Medicine, Department of Clinical Sciences, College of Veterinary Medicine North Carolina State University Raleigh North Carolina USA; ^3^ Comparative Pain Research and Education Center North Carolina State University Raleigh North Carolina USA; ^4^ Veterinary Medicine Research and Development Zoetis Inc Kalamazoo Michigan USA; ^5^ Veterinary Medicine Research and Development Zoetis Belgium SA Zaventem Belgium; ^6^ Thurston Arthritis Center UNC School of Medicine Chapel Hill North Carolina USA; ^7^ Center for Translational Pain Research, Department of Anesthesiology Duke University Durham North Carolina USA

**Keywords:** arthritis, client specific outcome measures, CSOM, degenerative joint disease, feline, feline musculoskeletal pain index, FMPI

## Abstract

**Background:**

Frunevetmab, a felinized antinerve growth factor monoclonal antibody, effectively decreases osteoarthritis (OA) pain in cats.

**Objective:**

To evaluate the efficacy of frunevetmab given at monthly intervals in a randomized, placebo‐controlled, parallel‐group, double‐blind superiority study.

**Animals:**

Two hundred seventy‐five client‐owned cats with naturally‐occurring OA pain and associated mobility impairment and disability.

**Methods:**

Randomized, placebo‐controlled, parallel‐group, double‐blind, superiority study. Following screening, cats received frunevetmab (nominal dose of 1.0 mg/kg, SC [effective dose range of 1.0‐2.8 mg/kg]) or placebo on days 0, 28, and 56. Outcome measures were owner questionnaires and veterinary physical and orthopedic evaluations at days 28, 56, and 84. Success/failure rates (and numbers needed treat, NNT) and change in scores (and standardized effect size, ES) were analyzed.

**Results:**

Frunevetmab (182) and placebo (93) treated cats were enrolled and received at least 1 treatment. Significant improvement with frunevetmab over placebo occurred at days 28 and 56 for the client specific outcome measures (CSOM) questionnaire (success rates and total scores [NNT of 9 and ES of 0.3 at day 56]); at days 28 and 56 for owner‐assessed global treatment response; and at days 56 and 84 for veterinarian‐assessed joint pain (ES of 0.18 at day 56). Adverse events did not differ between groups, except skin disorders which collectively occurred significantly more frequently in frunevetmab treated (32/182 cats) vs placebo (8/93 cats).

**Conclusions and Clinical Importance:**

Frunevetmab has the potential to address a critical gap in the treatment of pain because of osteoarthritis in cats.

**Abbreviations**
CMI: clinical metrology instrument
CSOM: client specific outcome measures
DJD: degenerative joint disease
FMPI: feline musculoskeletal pain index
NGF: nerve growth factor
OA: osteoarthritis

## 1 INTRODUCTION

Degenerative joint disease (DJD), and osteoarthritis (OA) are highly prevalent in cats with estimates indicating that 61% to 93% of all cats have radiographic DJD/OA.^1^, ^2^ One estimate suggests that 40% of all cats with radiographic DJD/OA experience related pain, and have clinical signs associated with this pain.^3^

In general, there is a lack of proven treatment options for cats with OA pain. While not approved in the United States, 2 NSAIDs (meloxicam and robenacoxib) are approved in parts of the world for the alleviation of chronic musculoskeletal pain in cats. Despite work showing that NSAIDs are effective for DJD/OA pain^4^ and can be used safely in many cats, including cats with chronic kidney disease,^5^, ^6^, ^7^ the mechanism of action of this class of drug creates adverse effects in some cats.^8^, ^9^ Additionally, it is clear that daily oral administration of drugs to cats, regardless of the formulation, is problematic for owners.^10^

Nerve growth factor (NGF) has an important role in nociceptor sensitization in a wide variety of both acute and chronic pain states including OA pain.^11^, ^12^, ^13^, ^14^ NGF appears to play a particularly important role in OA pain.^11^ There is strong evidence of robust analgesic effects of monoclonal antibodies (mAbs) directed against NGF.^15^, ^16^ These mAbs, directed against NGF, bind NGF therefore sequestering it and preventing it from interacting with its receptors.

Anti‐NGF mAbs are effective in dogs with OA,^17^, ^18^ with the first canine anti‐NGF mAb (bedinvetmab) recently approved for use in EU.^19^, ^20^ In human studies performed thus far, dose‐dependent efficacy was demonstrated in patients with moderate to severe pain associated with symptomatic knee or hip OA.^21^, ^22^, ^23^ Efficacy appeared greater than that observed with NSAIDs or opiates.^24^ In cats, proof‐of‐concept and pilot field studies demonstrated efficacy of a felinized anti‐NGF mAb (frunevetmab) in the treatment of DJD‐associated pain.^25^, ^26^

Despite considerable advances over the last 15 years in our understanding of how to measure chronic pain in cats,^27^ a profound placebo effect (up to 80%) is described in clinical chronic pain trials using subjective owner assessment that trends higher with longer duration studies.^28^

The objective of this study was to evaluate the efficacy of 3 SC administered doses of frunevetmab (recently approved as Solensia in the European Union) given at monthly intervals in a randomized, placebo‐controlled, parallel‐group, double‐blind superiority study across multiple veterinary practices. This study was conducted for registration purposes.

## 2 MATERIALS AND METHODS

### 2.1 Study design

This study was a multisite, randomized, placebo‐controlled, double‐blind study conducted according to Good Clinical Practice (GCP) guidelines^29^ that compared frunevetmab, a felinized anti‐NGF mAb, with placebo, in a superiority statistical analysis. All cats received 3 injections given 28‐days apart: Group 1 received frunevetmab SC; Group 2 received vehicle control SC. The primary outcome measures (efficacy measures) were owner assessments.

### 2.2 Animals and study sites

Client‐owned cats with naturally‐occurring OA pain and associated mobility impairment and disability were enrolled. Subjects were recruited at 21 veterinary clinics in the United States. A single licensed veterinarian served as the investigator at each site, whereas at least 1 additional person at the practice served as the treatment administrator. The investigator could serve as the examining veterinarian, or could designate an additional veterinarian. Owners and all personnel were blinded to the treatment.

### 2.3 Study timeline

**FIGURE 1:**
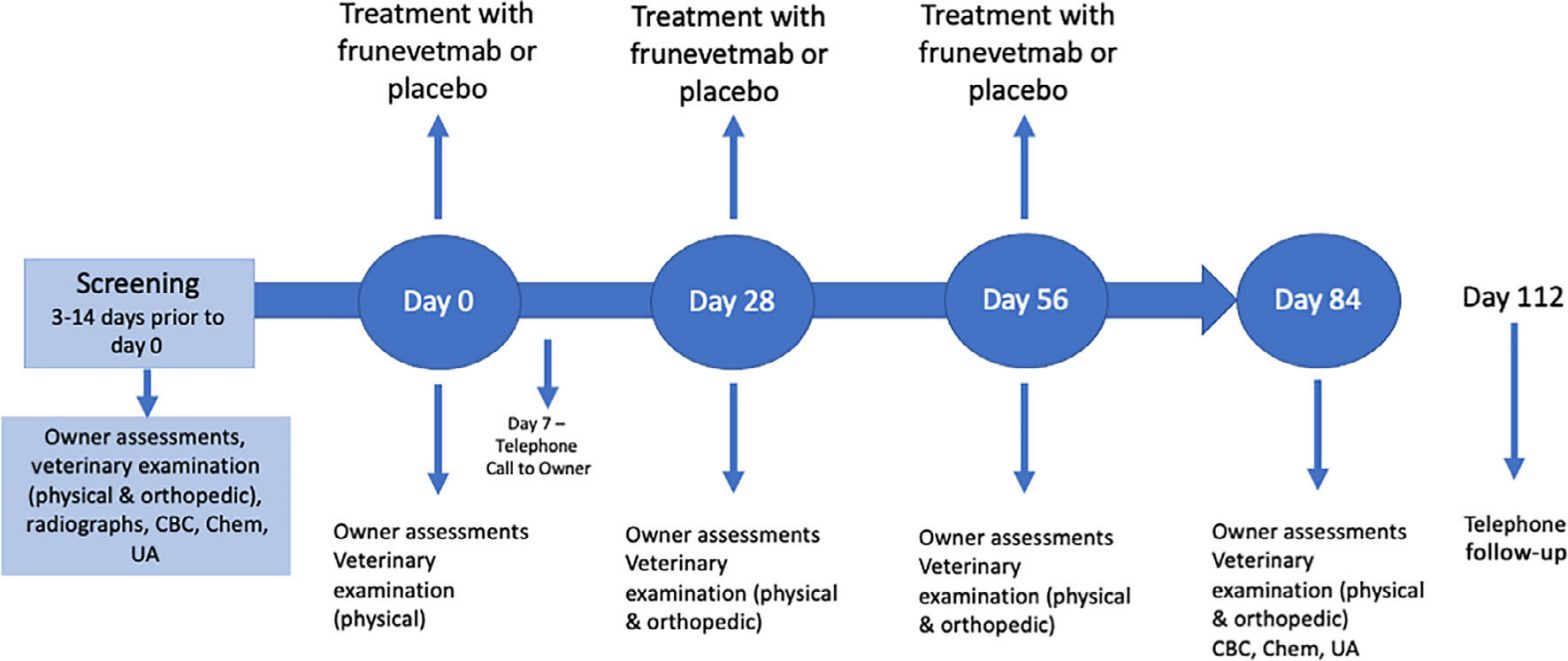
Schematic of study timeline

The overall study timeline is illustrated in Figure 1.

Cats were evaluated at screening (screening visit) with physical, neurologic, and orthopedic examinations, clinical pathology (complete blood count [CBC], serum chemistry, urinalysis) and radiographs for eligibility; owners completed clinical metrology instruments (CMIs; see section on outcome measures for details). Three to 14 days later, on day 0, owners visited the clinic completing the CMIs (baseline data); following a physical examination at day 0, cats were injected with either frunevetmab (SC) or placebo (SC). On day 7, owners received a telephone call to check on their cat's status. On days 28 and 56, owners visited the clinic and completed the CMIs, cats were evaluated with a physical and orthopedic examination, and were then given second (day 28) and third (day 56) injections of frunevetmab (SC) or placebo (SC). Each injection was given in a different location on an individual cat to allow appropriate site evaluations: injections sites were selected based on standard protocol at each veterinary practice. On day 84, owners visited the clinic, cats were evaluated (physical and orthopedic examination), clinical pathology (CBC, serum chemistry, urinalysis) was performed, and owners completed the CMIs. On day 112, owners received a telephone call to check on their cat's status.

### 2.4 Screening and inclusion/exclusion criteria

Cats of any sex and breed were eligible for screening if they were >6 months of age and ≥2.5 kg body weight. Each cat was required to: have clinical signs of OA noted by the owner; be in general good health, have a minimum client specific outcome measure (CSOM) score of ≥7 (on a scale of 3 = normal; 15 = highly impaired [see later for details]); and have radiographic evidence of OA in at least 2 joints deemed to be painful on the orthopedic examination. Radiographs of joints where pain was detected were performed, under sedation if necessary, at the screening visit, unless radiographs taken in the previous 12 months were available for review.

Cats who had stable chronic conditions (including renal disease up to and including IRIS stage 2) were able to be included. Cats were excluded from the study if they: were pregnant, lactating, or intended for breeding; had neurologic abnormalities or had undergone major surgery within the previous month or had a planned elective surgery during the study period that would interfere with assessments; had received previous treatment with any mAbs; had received vaccinations within 7 days before day 0; were receiving exclusionary medications or had other abnormal health findings, such as urinary tract infection or planned minor surgery (including dental procedures) that could interfere with evaluations. Withdrawal of exclusionary medications was allowed with the following time periods: 7 days before day 0 for NSAID medications, amantadine, tramadol and tricyclic antidepressants; 45 days for gabapentin and oral nutraceuticals (unless the cat had been administered these on a stable dose and interval regimen for >45 days and continued for study duration); 28 days for cyclosporin; 30 days for short‐acting steroids; and 60 days for Adequan (unless it had been administered for >60 days on a stable regimen and continued for study duration).

### 2.5 Efficacy outcome measures

The primary outcome measure was the CSOM; the primary effectiveness endpoint was treatment success day 56. Secondary effectiveness endpoints included CSOM success rate at days 28 and 84; owner global assessment, total CSOM scores, and orthopedic examination scores at days 28, 56, and 84. Cats could be administered any treatment for pain at any time as a rescue, but were then designated failures and exited the study.

### 2.6 Client specific outcome measures

The CSOM queries owners about their cat's ability to perform 3 individually‐tailored activities on a 5‐point scale from “No problem” to “Impossible.” The CSOM used in this study was a modification of a previously described and published system^25^, ^28^, ^30^, ^31^ (https://cvm.ncsu.edu/research/labs/clinical-sciences/comparative-pain-research/clinical-metrology-instruments/). The modification was in the assignment of scores to categories (see below). Activities were selected for each cat/owner dyad at the screening visit. Owners were allowed to change 1, 2, or all activities on day 0 after having observed their cat after the screening visit; following day 0, activities could no longer be changed. The same owner completed the CSOM at each timepoint. Ratings were converted to numerical scores from 1 (no problem) to 5 (impossible) and summed (score range of 3 [normal] to 15 [highly impaired]). A reduction from the day 0 score of ≥2 (with no increase in any individual score) was defined as a treatment success; number of successes/failures and total changes in scores from day 0 were compared between groups for days 28, 56, and 84.

### 2.7 Owner global assessment

On days 28, 56, and 84 the owner made a global assessment of the treatment's success in controlling clinical signs of OA in their cat. These were scored as: “Excellent”; “Good”; “Fair”; or “Poor” (Table S1). If a cat was withdrawn for lack‐of‐efficacy, the owner global assessment was recorded as “Poor.” The number and percentage in each category were compared between groups at days 28, 56, and 84.

### 2.8 Veterinary orthopedic assessment

Orthopedic examinations were performed at screening (baseline) and at days 28, 56, and 84. The protocol encouraged clinics to have the same veterinarian examine a cat at each timepoint. The veterinary orthopedic assessment followed the NC State Translational Research in Pain “Joint Evaluation and Scoring” form (source form shown in Table S2). However, scores for pain used a 1 to 5 scale was used instead of 0 to 4^30^ to facilitate subsequent statistical analysis. Scores for effusion, crepitus, and thickening were collected but not analyzed. Pain scores were summed to create a total pain score, and these scores were evaluated for changes from screening to days 28, 56, and 84.

### 2.9 Safety outcome measures

Safety assessments were made based on the findings of physical examinations performed on days 28, 56 and 84; injection site evaluations before and after treatments on days 0, 28, and 56, and once on day 84; clinical pathology (CBC, serum chemistry, urinalysis) on day 84 and as needed by individual cats; and adverse events reported by owners. At each visit, owners were asked whether any adverse events had occurred; between visits owners were asked to report any events they were concerned about immediately. All samples were analyzed by a central laboratory (IDEXX Laboratories, Sacramento, CA).

### 2.10 Treatments

Treatments were supplied in single‐use 2 mL glass vials each containing an extractable volume of 1 mL with a concentration of 7.0 mg/mL (±0.7 mg/mL) frunevetmab or no frunevetmab. Any cat weighing 2.5 to 7.0 kg received 1 vial (1.0 mL). Any cat >7.0 to 14.0 kg received 2 vials (2.0 mL). The nominal frunevetmab dose was 1.0 mg/kg, resulting in an effective dose range of 1.0 to 2.8 mg/kg with unit dosing.

### 2.11 Randomization, group assignment, and blinding

Cats were randomly assigned to treatment with frunevetmab or placebo based on order of entry into the study at a ratio of 2:1 (frunevetmab: placebo). Randomization was performed using the electronic data capture system (Prelude Dynamics, Austin, TX), using a “just‐in‐time” randomization procedure stratified within each study site. Active treatment and placebo were assigned codes, and assignment of the codes was not broken until statistical analysis. The Dispenser at the clinic knew the code, but not the underlying treatment assignment. Investigators, owners, and all other clinic staff were blinded to treatment assignment and treatment codes during and after the study. Dispenser, Investigator, or other clinic staff were permitted to administer the test articles.

### 2.12 Statistical analysis

Groups were compared for distribution of age and weight using *t*‐tests; sex distribution was compared using chi‐square analysis.

Sample size estimation was based on a pilot field study conducted under similar conditions.^26^ In that multisite field study, a CSOM success rate of 80.3% for frunevetmab and 44.7% for placebo was found on day 56 in 126 enrolled cats.^26^ These data indicated that an evaluable sample size of 60 frunevetmab treated and 30 placebo treated cats provided 90% power (alpha level = 0.05, 2‐sided) to detect a difference between treatment groups in CSOM success rate based on a change of 2 points on the CSOM.

Treatment success/failure analyses for CSOM and owner global assessment were evaluated using appropriate methods for binary or multinomial outcomes (GLIMMIX procedure in SAS) assuming a binomial distribution and logit link, or multinomial distribution and cumulative logit link. The models included treatment group as a fixed effect with site and treatment group by site interaction as random effects. Success/failure rates were used to calculate values for number needed to treat (NNT). NNT is considered a valuable method to compare the value of different treatments across studies. Simplistically, a NNT of “x” means “x” animals need to be treated for 1 additional animal to be designated a “success” as defined by the success/failure criteria in the study.

Total CSOM scores were subject to a repeated‐measures analysis of covariance (RMANCOVA), with the baseline total score as a covariate. Mean and SDs for outcome measures across placebo and treatment groups were used to calculate treatment efficacy over placebo ([treatment effect minus placebo effect]/[placebo effect] expressed as a percentage) on days 28, 56, and 84, and also to calculate standardized effect sizes over placebo (ES; mean of treatment group minus the mean of the control group/pooled SD) on day 56. Success rates based on different cut‐offs (improvement of 3, 4, or 5 on the CSOM) were also explored as a secondary, post hoc analysis.

Owner global assessment scores were evaluated by study day instead of as repeated measures given convergence issues with the latter approach. Total pain scores from the orthopedic evaluation were analyzed using an RMANOVA, using the baseline scores as a covariate. Mixed procedure ANCOVA was used to evaluate the influence of treatment on body weight, heart rate, respiratory rate or body temperature as measured on days 28, 56, and 84; and CBC, serum chemistry or urinalysis results on day 84. Screening values were included as the covariate. Adverse events were categorized, and incidence compared between groups using a Fisher's exact test. Testing was 2‐sided at the significance level *P* = .05.

## 3 RESULTS

**FIGURE 2:**
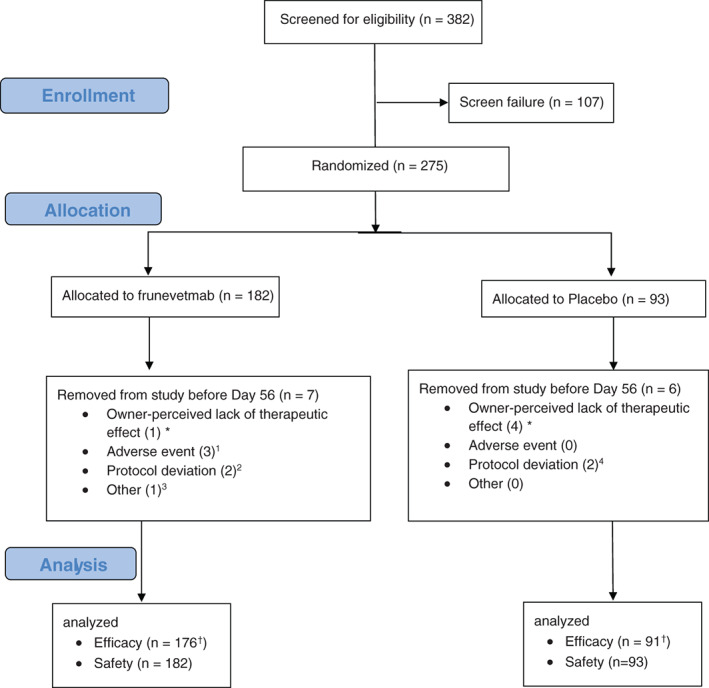
CONSORT flow diagram describing all the cases recruited to the study. ^†^Specifically for a priori CSOM day 56 analysis. *Cats withdrawn for owner perceived lack of effect were included in efficacy analysis as treatment failures. ^1^One cat removed day 12 for unrelated chronic cardiomyopathy, CKD, and chronic gastroenteritis; 1 cat removed day 22 for myocardial fibrosis and renal disease; 1 cat removed day 28 at owner's election because of worsening of intermittent vomiting. ^2^One cat removed day 13 for use of a prohibited medication, 1 cat removed day 25 because of owner's inability to complete assessments. ^3^One cat removed day 0 for receiving incorrect treatment. ^4^One cat had day 56 visit out of window, 1 cat was removed because of use of prohibited

A total of 382 cats were screened for enrollment, and 275 cases received at least 1 treatment (182 frunevetmab, 93 placebo) and were thus included in the assessment of treatment safety. Eight of these 275 cats were excluded from the assessment of treatment success on day 56 (6 frunevetmab: 3—adverse events; 2—protocol deviations; 1—other; 2 placebo: 2—protocol deviations; Figure 2, CONSORT flow diagram). The evaluation of effectiveness did not include cats withdrawn early for reasons that were unrelated to treatment, cats with adverse events that affected gait (other than those related to OA) and cats with significant study deviations such as use of a prohibited concomitant medication (eg, systemic anti‐inflammatory). Cats who were discontinued because of owner perceived lack of therapeutic effect were considered treatment failures for all subsequent evaluation days.

Demographic characteristics of each group of cats on day 0 enrolled in the study are shown in Table 1. There were no significant differences between the groups for age (*P* = .31), weight (*P* = .35), or sex distribution (*P* = .17). Characteristics of the enrolled cats reflect the characteristics of the target clinical study sample.^1^, ^3^, ^25^, ^30^, ^31^, ^32^

**TABLE 1:** Demographic characteristics of cats at day 0 receiving at least one treatment, in each treatment group

Parameter		Frunevetmab (N = 182)	Placebo (N = 93)	*P*‐value
Age (years)	Mean (±SD)	13.14 (3.21)	12.72 (3.16)	.31
	Median (Min, Max)	13.29 (1.58, 22.42)	13.17 (3.5, 18.17)	
Weight (kg)	Mean (±SD)	5.35 (1.55)	5.54 (1.73)	.35
	Median (Min, Max)	5.28 (2.49, 10.21)	5.44 (2.68, 11.39)	
Sex	Female spayed	106 (58.24%)	46 (49.46%)	.16
	Male castrated	76 (41.76%)	47 (50.54%)	
CSOM score	Mean (±SD)	11 (1.9)^a^	11.4 (1.8)	.2
	Median (Min, Max)	11 (7, 15)	11 (7, 15)	

a CSOM score n = 178.

Safety was assessed through the review of treatment‐emergent adverse events, clinical pathology results and physical examinations. All cats who received at least 1 dose of frunevetmab or placebo were included in the safety analysis.

### 3.1 Client specific outcome measures

**FIGURE 3:**
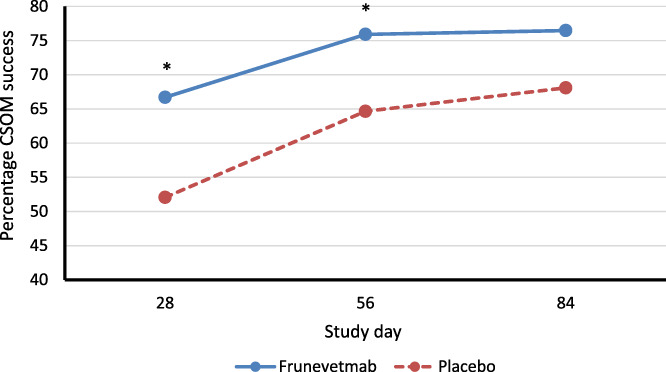
Percentage of cats with treatment success, where success is defined as a reduction in the client specific outcome measure (CSOM) score of ≥2 for any individual cat. The number of successes in each group were compared using appropriate methods for binary outcomes (GLIMMIX procedure in SAS) at study days 28, 56, and 84. *P < .05 indicating a significantly greater percentage of cats in the frunevetmab group were designated successes

CSOM scores were not different between the groups at the start of the study, day 0 (*P* = .2). A significantly greater percentage of cats in the frunevetmab‐treated group achieved treatment success compared to placebo at the primary efficacy endpoint, day 56:75.91% vs 64.65% (*P* = .03; Figure 3). Additionally, a significantly greater percentage of cats in the frunevetmab‐treated group achieved treatment success compared to placebo on day 28:66.70% vs 52.06% (*P* = .02). On day 84, cats in the frunevetmab‐treated group had a numerically higher success rate (76.47% success) than cats in the placebo‐treated group (68.09%), but this was not significant (*P* = .08). These data, and confidence limits are given in Table 2. The treatment effect over placebo, expressed as a percentage, was 28%, 17%, and 12% at days 28, 56, and 84, respectively. The NNT calculated from day 56 data is 9.

**TABLE 2:** Summary of treatment success^a^ for CSOM at days 28, 56, and 84

	Group	N	% success^b^	95% confidence interval	*P*‐value
Day 28	Frunevetmab	178	66.7	59.64, 73.09	.02*
	Placebo	93	52.06	41.84, 62.11	
Day 56	Frunevetmab	176	75.91	69.06, 81.65	.03*
	Placebo	91	64.65	53.99, 74.02	
Day 84	Frunevetmab	167	76.47	69.57, 82.21	.08
	Placebo	89	68.09	57.60, 77.01	

Abbreviation: N, number of animals.

^a^ Model‐derived least square means.

^b^ Back‐transformed from the logit transformation used in the statistical analysis.

* significant *p*‐value.

Success rates based on varying cut‐offs for improvement are presented in Tables 3 and 4. Since inclusion criteria were set at a Total CSOM score of 7, not all cats had the potential to improve by 5 points, since the lowest score achievable was 3 (a score of 1 for each category represented a healthy cat). Overall, the frunevetmab cats had significantly higher CSOM success rates than placebo.

**TABLE 3:** Treatment success based on varying cut‐offs for the decrease in the total client specific outcome measures scores at day 56

	Decrease in total CSOM score vs day 0 (cut‐off for treatment success)			
% of cats with treatment success	≥2	≥3	≥4	≥5^a^
Placebo group	64.65	52.77	39.40	29.74
Frunevetmab group	75.91	69.29	58.32	43.61
*P*‐value	0.031*	0.018*	0.012*	0.032*
Treatment effect^b^	11.26%	16.52%	18.92%	13.87%
NNT	8.9	6.1	5.3	7.2

*Note*: Statistical comparison (*P*‐value) and treatment effect is also shown for each cut‐off.

Abbreviation: NNT, number needed to treat.

^a^ Frunevetmab n = 8 and placebo n = 2 could not be included in the analysis because CSOM scores were <8 at baseline, meaning they could not improve by ≥5 (lowest score possible on CSOM was 3).

^b^ Calculated using the formula [treatment effect minus placebo effect]/[placebo effect] expressed as a percentage.

* Significant *P*‐value.

**TABLE 4:** Treatment success based on cut‐offs of 3 and 4 for the decrease in the client specific outcome measures scores tabulated for each assessment timepoint

Threshold change in CSOM to qualify as success	Study day	Placebo	Frunevetmab	*P*‐value
≥3	Day 28	41.94%	56.18%	.01*
	Day 56	52.75%	69.32%	.02*
	Day 84	59.55%	68.86%	.16
≥4	Day 28	27.96%	41.57%	.02*
	Day 56	39.56%	57.95%	.01*
	Day 84	49.44%	59.28%	.13

*Note*: Statistical comparison (*P*‐value) also shown for each cut‐off.

^*^ Significant *P*‐value.

Overall (across all posttreatment time points), total CSOM scores were significantly lower in the frunevetmab treated group compared to the placebo treated cats (*P* = .03). Total CSOM scores were numerically lower in the frunevetmab treated cats at all posttreatment time points, significantly so on days 28 (*P* = .03) and 56 (*P* = .02) (Table 5). The standardized ES at day 56 was 0.3.

**TABLE 5:** Summary of total CSOM scores, showing least squared means (SEM) for each group, at each assessment time point

Day	Least squares means (SEM)		*P*‐value
	Frunevetmab	Placebo	
28	8.13 (0.21)	8.83 (0.28)	**.03**
56	7.08 (0.23)	7.93 (0.31)	**.02**
84	6.76 (0.23)	7.46 (0.32)	.06
Pooled across days	7.33 (0.21)	8.07 (0.28)	**.03**

### 3.2 Owner global assessment

The distribution of the overall owner assessment favored the frunevetmab group at each time point, reaching statistical significance on days 28 and 56 (*P* = .03 and *P* = .04, respectively), but not for day 84 (Table 6).

**TABLE 6:** Summary of the owner global assessment categories (percentage [%] of cats in each category)

Study day	Group	N	% excellent	% good	% fair	% poor	*P*‐value
28	Frunevetmab	178	6.18	33.15	44.38	16.29	**.02**
	Placebo	92	2.17	28.26	40.22	29.35	
56	Frunevetmab	172	14.53	44.77	23.84	16.86	**.04**
	Placebo	87	4.6	43.68	27.59	24.14	
84	Frunevetmab	164	25	39.63	20.12	15.24	.1
	Placebo	83	16.87	40.96	16.87	25.3	

Abbreviations: N, number of animals; %, percentage.

### 3.3 Veterinary orthopedic assessment

The overall treatment effect on change in total pain scores was significant (*P* = .04), with lower scores in the frunevetmab group at each time point, statistically lower on days 56 (*P* = .02) and 84 (*P* = .04), but not on day 28 (*P* = .3; Table 7). The standardized ES at day 56 was 0.18.

**TABLE 7:** Change in total pain scores from screening (least squares [LS] means and SEM) from orthopedic evaluation by veterinarians, and statistical comparisons at each time point

Study day	Group	N	LS means (SEM)	*P*‐value
28	Placebo	92	−4.7 (0.6)	.3
	Frunevetmab	178	−5.3 (0.6)	
56	Placebo	89	−5.0 (0.7)	.02
	Frunevetmab	175	−6.4 (0.6)	
84	Placebo	83	−5.3 (0.7)	.04
	Frunevetmab	166	−6.6 (0.6)	
Overall	Placebo	264	−5.0 (0.6)	.04
	Frunevetmab	519	−6.1 (0.5)	

Abbreviation: N, number of animals.

### 3.4 Safety

There were 354 adverse events (AE) described in 158 cats (113 of 182 cats who received frunevetmab and 45 of 93 cats who received placebo). AEs reported in >2% of cats in either group are detailed in Table S3. Overall, similar signs were reported in both groups and the majority of AEs/serious AEs (SAEs) reported were classified as being unlikely to be related to treatment. Digestive tract disorders including emesis and diarrhea were the most frequently reported AEs, with similar occurrences in both treatment groups. When taken collectively as alopecia, dermatitis/eczema, pruritus, and skin disorders (not otherwise specified, NOS), skin lesions (NOS) and bacterial skin infections, there were higher percentages of AEs in the frunevetmab treated cats compared to placebo treated cats (*P* < .0001—see Table S3 for details). Out of the 32 frunevetmab‐treated cats experiencing a skin condition, 13 cats did not require treatment. The remaining 19 cats received standard medical treatment (topical or systemic treatment with antimicrobials and antihistamines, and topical corticosteroid‐containing anti‐inflammatories), and the skin conditions resolved or improved. Cats who received anti‐inflammatories before day 84 (with the exception of those that were for otic or ophthalmic use) were removed from the study, as per protocol (see Figure 2). The skin conditions of 3 frunevetmab‐treated cats did not improve during the study: 2 cats with lumps and 1 cat with alopecia. These 3 cats did not require any medical treatment.

Ten cats experienced adverse events that were considered serious, including 7 frunevetmab treated cats (of 182) and 3 placebo treated cats (of 93). Of these events, 4 resulted in euthanasia for frunevetmab treated cats and 1 of the placebo‐treated cats died. In frunevetmab treated cats, 3 were diagnosed with cancers (1 on day 78; 2 were euthanized on days 61 and 72); 2 were diagnosed with chronic renal failure (1 considered mild on histopathology, the other considered end stage) and cardiac pathology (on day 12 and on day 22, both euthanized); 1 was diagnosed with urinary tract infection (day 84); 1 diagnosed with inflammatory myopathy (day 26). In placebo treated cats, 1 was diagnosed with destructive rhinitis (day 58); 1 observed with convulsions—no diagnosis made (day 84); and 1 with hypertrophic cardiomyopathy (died, day 84). The relation of these serious adverse events to treatment was considered unlikely except the case of inflammatory myopathy which was considered unknown. Cats with serious AEs exited the study, but efficacy data were retained for assessment of prior time points.

There were no statistically significant or clinically meaningful effects of treatment on body weight, heart rate, respiratory rate or body temperature as measured on days 28, 56, and 84. At the day 112 telephone follow up (~60 days after the last injection of frunevetmab/placebo for the 162 frunevetmab cats and 81 placebo cats who completed the study), owners of 51 (39 frunevetmab; 12 placebo) cats reported abnormal findings, the majority related to worsening/returning clinical signs associated with previously diagnosed OA (23/39 [59.0%] in the frunevetmab group; 5/12 [41.7%] in the placebo group. One abnormal injection site reaction was reported in 1 cat in the placebo group when presented for day 56 evaluation. The cat was reported to have a 4 to 5 mm subcutaneous mobile lump at the injection site (caudo‐lateral stifle). It was aspirated and the cytology findings were consistent with lipoma. Fourteen incidents of pain upon injection were reported for 11 cats, 9 occurrences in 7 frunevetmab treated cats (of 182), and 5 in 4 placebo treated cats (of 93).

There were no clinically meaningful differences between the treatment groups in CBC, serum chemistry or urinalysis results. Mean corpuscular hemoglobin (MCH) values in the frunevetmab group were significantly (*P* value just less than .05) lower than values in the placebo group on day 84 but both group means were within the normal reference interval and were not considered clinically relevant. Selected hepatic, renal, urinalysis, and hematological results are shown in Table S4.

## 4 DISCUSSION

In this study, frunevetmab, a felinized anti‐NGF mAb, administered 3 times at 28‐day intervals, at a dose range of 1.0 to 2.8 mg/kg by SC injection, demonstrated superior efficacy to placebo in decreasing mobility impairment, disability, and pain associated with OA in cats as measured across several different dimensions. Efficacy was measured using a validated, owner‐completed questionnaire (CSOM), an owner assessment of global treatment response, and veterinarian‐assessed joint pain. Frunevetmab treated cats showed greater positive responses than placebo at all time points across all outcome measures, although there was some variability in the timepoints at which statistical significance was reached. At later time points, the lack of statistical significance could have been because of the increasing placebo effect over the course of the study, since the success rate of frunevetmab treated cats did not appear to decrease throughout the study (66.7% to 75.91% to 76.47% at days 28, 56, and 84, respectively). The placebo response was consistent with studies of analgesics in cats,^28^ although on the higher end of the spectrum (~52%‐68% for CSOM success). There were no apparent differences in measured safety between placebo and frunevetmab, except for dermatological AEs that occurred more frequently with frunevetmab.

The CSOM is an owner‐based assessment of the impact of DJD or OA pain in cats, and considered valid if used properly.^27^ Chronic pain impacts behavior, and behavior is dramatically altered when cats are seen in the veterinary clinic^33^; therefore the best persons to assess behavior are owners, evaluating cats in the home environment.^27^ Success‐failure was calculated at each time point, based on a change (improvement) in the total CSOM score of 2 or greater. Given that a total CSOM score of 3 in this study equated to “normal” and the median initial score was ~11.0, then the maximum change possible in this study was, on average, 8 (a decrease in disability from 11 to 3). Therefore, with treatment success defined as a change of “2,” this equated to a 25% reduction in disability [(2/8) * 100]. This is considered to be on the low side of clinically meaningful in human medicine.^34^, ^35^ When the cut‐offs for success were increased to 3, or 4 (representing a 37% and 50% decrease in disability, respectively), statistical superiority of frunevetmab over placebo at days 28 and 56 was maintained (Table 4). A 50% decrease in pain/disability is considered clinically meaningful.^34^, ^35^ Success‐failure outcomes can be converted to numbers needed to treat (NNT), an accepted way of defining clinical utility of a therapeutic where lower numbers indicate greater effectiveness. For frunevetmab, the NNT at day 56 was 8.9, and interestingly this dropped to 6.1 and 5.3 as the criteria for success were increased to a 37% and 50% reduction in disability, respectively (Table 3), reflecting a greater separation from placebo as the threshold for success is increased. The NNT used for NSAIDs in humans is between 3 and 13, depending on the criteria for success.^36^ There are no data from studies of chronic pain in cats with which to compare these values, except for 1 previous study of frunevetmab, where the NNT at day 56 (after 2 doses of frunevetmab at monthly intervals) was 3.^26^ The higher NNT in the present study appears to be because of a higher caregiver placebo effect in the current study.

Standardized effect size is another way to compare efficacy across therapeutics, with higher numbers indicating greater efficacy. In the present study, based on CSOM scores, an ES of 0.3 was found at day 56, which was lower than that seen in the previous frunevetmab studies where the ES at day 56 was 0.65.^26^ An ES of 0.3 is identical to that expected from daily NSAID administration in humans,^37^, ^38^ and comparable to anti‐NGF mAbs therapies in humans. ES for anti‐NGF mAbs in humans have varied, depending on the dose, from −0.15 to 0.7,^16^ and if only Phase III studies are considered, the range is 0.25 to 0.61.^16^ In the pilot proof of concept efficacy study with frunevetmab for cats, the ES (at 3 weeks after a single dose) was 0.74.^25^

The strength of the placebo effect in clinical trials of treatments for chronic musculoskeletal pain in cats^28^ makes it difficult to detect true treatment effects. The placebo effect in studies of cats appears much higher than in studies involving dogs.^39^ The reason for this is not fully characterized, but could relate to a relative lack of our understanding how to measure improvement and the complex relationship between cats and their owners.^40^ The caregiver placebo effect in the current study was greater than that seen in earlier studies with frunevetmab,^25^, ^26^ and this could have contributed to a smaller margin of difference between placebo and treatment in the owner assessment in the current study compared to previous studies at day 56. The larger placebo effect could have occurred for a number of reasons. Expectation bias^41^, ^42^ because of veterinarian expectations, owner expectations, or both based on the positive initial proof of concept study results^25^ could have contributed. The knowledge of these results could have inflated the placebo effect. Other factors including the larger study size and longer study duration could also have contributed.^43^ There are few pain trials in veterinary medicine that have been conducted over extended periods of time, and thus there is a poor understanding of how factors such as participant (owner) fatigue influence subjective results over time. In human clinical trials, the placebo effect does not diminish over time,^42^ and in the pilot proof of concept study with frunevetmab, the placebo rate was maintained out to the end of the study (63 days after treatment).^25^ A combined analysis of the data from all the frunevetmab trials would provide a better estimate of the clinical utility rather than relying on the results of single trial.

Pain impacts multiple dimensions (physical, behavioral, affective, among others)^27^; not all of these will be equally improved by a therapeutic, and improvement is unlikely to be equal at all time points. This might have been another factor that contributed to the varying statistical efficacy at different time points across outcome measures (and indeed, is a factor in all pain studies).

To get a sense of the reduction in joint pain based on the veterinary examination, the joint pain scoring system (where a total pain score of 21 represents a cat without any OA pain) can be adjusted to calculate the percentage change. The mean percentage change in the joint pain scores in the frunevetmab treated cats was 40.6%, 49.5%, and 50.0% at day 28, 56, and 84, respectively, whereas in the placebo group the change was a 31.6%, 37.4%, and 36.5% reduction on the same days, respectively. As noted, a 50% decrease in pain/disability is considered clinically meaningful^34^, ^35^ and notable in reaching statistical significance given that measuring joint pain is difficult in clinical situations.

Many cats were enrolled that had concurrent stable disease—expected in a sample of cats with a median age of 13 years. The inclusion/exclusion criteria ruled out apparent, confounding disease states, but it is possible undetected diseases were present. However, it is unknown what the impact of any undetected disease were—for example, objectively measured activity is not different in cats with subclinical HCM vs controls, despite HCM cats having lower (not significantly) systolic BP.^44^ Measures of safety (eg, CBC, serum chemistry, and urinalysis) and adverse events (based on veterinary examination and owner reports) were no different between placebo and frunevetmab treated cats, except for collective skin‐related adverse events (alopecia, dermatitis/eczema, pruritus and other skin disorders, lesions and bacterial infections; Table S4). Pruritus might have contributed to some occurrences of alopecia. Although speculative, the pruritus could reflect the human condition of paraesthesias (abnormal sensation), a reported adverse effect with anti‐NGF therapy in humans.^45^ The reason for the focal alopecia seen in the other frunevetmab treated cats is unclear. In most cats the skin conditions had some primary nidus (eg, traumatic injuries, flea infestation) or relationship to a preexisting condition (eg, history of allergic dermatitis). However, further work is needed to better understand the reasons for skin‐related adverse events. Since frequencies of dermatological AEs after each dosing were similar and resolution was seen with continued dosing, it does not seem that repeated administration increased the risk of a cat developing a skin condition. These events were mild, did not require treatment or resolved with standard treatments.

### 4.1 Conclusion and clinical relevance

Positive treatment effects from baseline to 84 days were seen in cats with OA pain and associated mobility impairment and disability, with the administration of 3 monthly doses of frunevetmab, a felinized anti‐NGF antibody. No adverse events attributable to the frunevetmab were seen except for skin‐related adverse events, specifically dermatitis, pruritus, and alopecia. As an injectable therapy providing long‐lasting effectiveness, frunevetmab precludes the need to medicate cats orally,^10^ and provides an attractive option for the treatment of chronic pain in cats.

## CONFLICT OF INTEREST DECLARATION

Jamie A. E. Myers, Jezaniah‐Kira S. Tena, Csilla Becskei, and Dawn M. Cleaver are employed by Zoetis; Margaret E. Gruen and B. Duncan X. Lascelles have received honoraria for continuing education lectures sponsored by Zoetis; Margaret E. Gruen and B. Duncan X. Lascelles are paid consultants for Zoetis.

## OFF‐LABEL ANTIMICROBIAL DECLARATION

Authors declare no off‐label use of antimicrobials.

## INSTITUTIONAL ANIMAL CARE AND USE COMMITTEE (IACUC) OR OTHER APPROVAL DECLARATION

This study was reviewed by the Zoetis Ethical Review Board. The study was performed according to the US and EC regulations/Directives and international guidance for new animal drugs (New Animal Drug for Investigational Use Exempt from Section 512(A) of the Food, Drug and Cosmetic Act, US Code of Federal Regulations (CFR), 21CFR511.1; Good Clinical Practice Guideline 9, VICH, June 2000; and FDA‐CVM GFI #85, May 2001). Informed, written owner consent was required before any procedures were performed with an individual cat.

## HUMAN ETHICS APPROVAL DECLARATION

Authors declare human ethics approval was not needed for this study.

## Supporting information


**Table S1** Owner global assessment evaluation scale. On days 28, 56, and 84 owners were asked to indicate the overall response to treatment (category), and were shown the descriptors for each category indicated in the table. The number and percentage of each category were compared between groups at days 28, 56, and 84

**Table S2** NC state translational research in pain (TRiP) feline musculoskeletal pain scoring system

**Table S3** Adverse events by VeDDRA (Veterinary Dictionary for Drug Regulatory Activities) system organ class and preferred term reported in >2% of cats in either treatment group

**Table S4** Selected hematological, serum chemistry, and urinalysis parameters at baseline (screening) and at the end of the study (day 84) in cats treated with frunevetmab or placebo. Means and SDs are reported, as are the number of cases that were above (“high”) or below (“low”) the reference range (provided in units used in the study). *n = 93(screening), n = 85 (day 84); for USG n = 90 (screening), n = 79 (day 84) **n = 182 (screening), n = 165(day 84); for USG n = 175 (screening), n = 149 (day 84)
