# Supplementary material for: Frunevetmab, a felinized anti‐nerve growth factor monoclonal antibody, for the treatment of pain from osteoarthritis in cats
Source: J Vet Intern Med. 2021 Nov 1;35(6):2752–62. doi: 10.1111/jvim.16291 (PMC8692178; doi:10.1111/jvim.16291)
Supplement: Supplementary file 1 — Table S1 Owner global assessment evaluation scale. On days 28, 56, and 84 owners were asked to indicate the overall response to treatment (category), and were shown the descriptors for each category indicated in the table. The number and percentage of each category were compared between groups at days 28, 56, and 84 [file JVIM-35-2752-s002.pdf]

Supplementary Table 1. Owner Global Assessment evaluation scale. On Days 28, 56 and 84 owners were asked to indicate the overall response to treatment (category), and were shown the descriptors for each category indicated in the table. The number and percentage of each category were compared between groups at Days 28, 56 and 84.

Category	Descriptor
Excellent	Clinical signs of OA were eliminated or reduced to an inconsequential level.
Good	Clinical signs of OA were substantially [at least 50 %] reduced.
Fair	Clinical signs of OA were minimally [less than 50 %] reduced.
Poor	Clinical signs of OA were unaffected by therapy.
