# Supplementary material for: Frunevetmab, a felinized anti‐nerve growth factor monoclonal antibody, for the treatment of pain from osteoarthritis in cats
Source: J Vet Intern Med. 2021 Nov 1;35(6):2752–62. doi: 10.1111/jvim.16291 (PMC8692178; doi:10.1111/jvim.16291)
Supplement: Supplementary file 2 — Table S2 NC state translational research in pain (TRiP) feline musculoskeletal pain scoring system [file JVIM-35-2752-s004.pdf]

Supplementary Table 2. NC State Translational Research in Pain (TRiP) Feline Musculoskeletal Pain Scoring System

Joint Evaluation and Scoring.

The following table was completed during the orthopedic examination based on the examiner's subjective assessment of each joint following palpation and passive extension, flexion and other manipulations as deemed appropriate. *Note: in the study reported here, the scoring was modified to be on a 1-5 (pain) or 1-3 (crepitus, effusion, thickening) scale to facilitate statistical analysis.*

R Forelimb	Pain (0-4)	Crepitus (0-2)	Effusion (0-2)	Thickening (0-2)
Feet				
Carpus				
Elbow				
Shoulder				
**R Hindlimb**	**Pain (0-4)**	**Crepitus (0-2)**	**Effusion (0-2)**	**Thickening (0-2)**
Feet				
Hock				
Stifle				
Hip				
**L Forelimb**	**Pain (0-4)**	**Crepitus (0-2)**	**Effusion (0-2)**	**Thickening (0-2)**
Feet				
Carpus				
Elbow				
Shoulder				
**L Hindlimb**	**Pain (0-4)**	**Crepitus (0-2)**	**Effusion (0-2)**	**Thickening (0-2)**
Feet				
Hock				
Stifle				
Hip				

Spinal Column	Pain (0-4)
Cervical	
Thoracic	
T-L	
Lumbar	
L-S	

Key to scoring

Pain scale based on palpation

0 **No resentment**; normal amount of movement or wriggling
1 **Mild withdrawal**; mildly resists
2 **Moderate withdrawal**; body tenses; **may orient** to site; **may vocalize** / increase in vocalization
3 **Orients to site**; **forcible withdrawal** from manipulation; may vocalize or hiss or bite
4 **Tries to escape / prevent manipulation**; **bite/hiss**; marked guarding of area

Crepitus, Effusion, Thickening: none (0); slight-moderate (1); significant-severe (2)

NC STATE 9 North Carolina State University, Translational Research in Pain (TRiP)
