# Supplementary material for: Frunevetmab, a felinized anti‐nerve growth factor monoclonal antibody, for the treatment of pain from osteoarthritis in cats
Source: J Vet Intern Med. 2021 Nov 1;35(6):2752–62. doi: 10.1111/jvim.16291 (PMC8692178; doi:10.1111/jvim.16291)
Supplement: Supplementary file 3 — Table S3 Adverse events by VeDDRA (Veterinary Dictionary for Drug Regulatory Activities) system organ class and preferred term reported in >2% of cats in either treatment group [file JVIM-35-2752-s001.pdf]

Supplementary Table 3: Adverse events by VeDDRA (Veterinary Dictionary for Drug Regulatory Activities) system organ class and preferred term reported in > 2% of cats in either treatment group[1]

System Organ Class	Preferred Term	Frunevetmab	Placebo
Digestive tract disorders	Emesis	24/182 (13.2%)	10/93 (10.8%)
	Diarrhea	12/182 (6.6%)	5/93 (5.4%)
	Gingival disorder	5/182 (2.7%)	0/93 (0.0%)
	Tooth disorder	4/182 (2.2%)	2/93 (2.2%)
	Intestinal stasis	0/182 (0.0%)	2/93 (2.2%)
Renal and urinary disorders	Renal insufficiency	12/182 (6.6%)	4/93 (4.3%)
	Urine abnormalities	8/182 (4.4%)	2/93 (2.2%)
Systemic disorders	Anorexia	12/182 (6.6%)	4/93 (4.3%)
	Lethargy	11/182 (6.0%)	3/93 (3.2%)
	Dehydration	8/182 (4.4%)	0/93 (0.0%)
	Weight loss	6/182 (3.3%)	5/93 (5.4%)
Skin and appendages disorders[1, *]	Dermatitis and eczema	11/182 (6.0%)	1/93 (1.1%)
	Alopecia	10/182 (5.5%)	2/93 (2.2%)
	Pruritus	7/182 (3.8%)	0/93 (0.0%)
	Skin disorders NOS[2]	6/182 (3.3%)	1/93 (1.1%)
	Skin lesions NOS[2]	5/182 (2.7%)	0/93 (0.0%)
	Bacterial skin infection	4/182 (2.2%)	1/93 (1.1%)
	Skin and/or appendage neoplasm NOS[2]	3/182 (1.6%)	3/93 (3.2%)
Musculoskeletal disorders	Lameness[3]	8/182 (4.4%)	2/93 (2.2%)
	Myopathy	1/182 (0.5%)	2/93 (2.2%)
Application site disorders	Injection site pain	7/182 (3.8%)	4/93 (4.3%)
Psychological disorders	Abnormal behavior	7/182 (3.8%)	1/93 (1.1%)
Unclassifiable event	Unclassifiable adverse event	6/182 (3.3%)	3/93 (3.2%)
Ear and labyrinth disorders	Otitis externa	4/182 (2.2%)	0/93 (0.0%)
Eye disorders	Eye disorder NOS[2]	4/182 (2.2%)	3/93 (3.2%)
Respiratory tract disorders	Sneezing	2/182 (1.1%)	3/93 (3.2%)
	Rhinitis	1/182 (0.5%)	3/93 (3.2%)
Blood and lymphatic system disorders	Leukocytosis	1/182 (0.5%)	3/93 (3.2%)
Behavioral disorders	Anxiety	0/182 (0.0%)	2/93 (2.2%)
Cardio-vascular system disorders	Murmur	0/182 (0.0%)	2/93 (2.2%)
Neurological disorders	Convulsion	0/182 (0.0%)	2/93 (2.2%)

* Higher percentages of AEs in this category in the frunevetmab treated cats compared to placebo treated cats (P<0.0001). No relationship between AE reports and study day

1 If a cat experienced the same event more than once, then only the first occurrence was tabulated.

2 Not Otherwise Specified (NOS)

3 New lameness or worsening of previous lameness.
