# Supplementary material for: Frunevetmab, a felinized anti‐nerve growth factor monoclonal antibody, for the treatment of pain from osteoarthritis in cats
Source: J Vet Intern Med. 2021 Nov 1;35(6):2752–62. doi: 10.1111/jvim.16291 (PMC8692178; doi:10.1111/jvim.16291)
Supplement: Supplementary file 4 — Table S4 Selected hematological, serum chemistry, and urinalysis parameters at baseline (screening) and at the end of the study (day 84) in cats treated with frunevetmab or placebo. Means and SDs are reported, as are the number of cases that were above (“high”) or below (“low”) the reference range (provided in units used in the study). *n = 93(screening), n = 85 (day 84); for USG n = 90 (screening), n = 79 (day 84) **n = 182 (screening), n = 165(day 84); for USG n = 175 (screening), n = 149 (day 84) [file JVIM-35-2752-s003.pdf]

Supplementary Table 4. Selected hematological, serum chemistry, and urinalysis parameters at baseline (screening) and at the end of the study (Day 84) in cats treated with frunevetmab or placebo. Means and standard deviations (SD) are reported, as are the number of cases that were above ('High') or below ('Low') the reference range (provided in units used in the study). * n= 93(screening), n=85 (Day 84); for USG n=90 (screening), n=79 (Day 84) **n=182 (screening), n=165(Day 84); for USG n=175 (screening), n=149 (Day 84)

	Placebo*				Frunevetmab**				P-value	Reference Interval
Variable	Mean	SD	Low	High	Mean	SD	Low	High		
Hematocrit - screening	38.0	6.0	5	0	38.1	6.1	7	1	0.8073	28.2-52.7%
Hematocrit - Day 84	39.2	5.6	2	1	39.2	6.0	6	1		
ALP - screening	25	11	1	1	24	13	12	2	0.5994	12-59 U/L
ALP – Day 84	26	13	2	2	25	15	12	4		
ALT - screening	54	31	4	1	55	29	2	2	0.1746	27-158 U/L
ALT - Day 84	62	58	4	3	55	28	4	3		
BUN - screening	30	8	0	14	31	9	2	35	0.0545	16-37 mg/dL
BUN - Day 84	31	9	0	16	32	10	0	46		
Creatinine - screening	1.5	0.4	4	3	1.5	0.4	3	4	0.2192	0.9-2.5 mg/dL
Creatinine - Day 84	1.5	0.5	2	1	1.6	0.5	7	7		
SDMA - screening	12	4	0	25	13	4	0	52	0.6381	0-14 ug/dL
SDMA – Day 84	13	4	0	23	14	5	0	52		
Total Protein - screening	7.2	0.6	1	1	7.1	0.5	10	0	0.5127	6.3-8.8 g/dL
Total Protein - Day 84	7.4	0.6	1	3	7.3	0.5	6	0		
Albumin - screening	3.2	0.3	3	0	3.1	0.3	5	0	0.1157	2.6-3.9 g/dL
Albumin - Day 84	3.1	0.3	3	0	3.1	0.3	5	0		
USG - screening	1.03	0.01			1.03	0.01			N/A	N/A
USG - Day 84	1.03	0.02			1.03	0.02				
